# Non-Invasive Localization of Epileptogenic Zone in Drug-Resistant Epilepsy Based on Time–Frequency Analysis and VGG Convolutional Neural Network

**DOI:** 10.3390/bioengineering12050443

**Published:** 2025-04-23

**Authors:** Yaqing Liu, Yalin Wang, Tiancheng Wang

**Affiliations:** 1 The Second Hospital and Clinical Medical School, Lanzhou University, Lanzhou 730030, China; 2 School of Information Science and Engineering, Lanzhou University, Lanzhou 730000, China; 3 Key Laboratory of Special Functional Materials and Structural Design, Ministry of Education, Lanzhou University, Lanzhou 730000, China

**Keywords:** drug-resistant epilepsy, time–frequency analysis, non-invasive localization, epileptogenic zone, VGG-16

## Abstract

The mainstream method for treating drug-resistant epilepsy (DRE) is surgical resection of the epileptogenic zone. Non-invasive automatic localization of epileptogenic zone can be used to guide electrode implantation and improve the effectiveness and safety of neurosurgical treatments. Previous researchers have proposed a range of methods for this purpose, but these suffer from limits such as unclear post-operative outcomes, invasiveness, limited data volume, and single DRE type. This study constructed a non-invasive epilepsy localization method, integrating sLORETA source imaging, time–frequency analysis, and Visual Geometry Group (VGG-16) deep learning. Firstly, 16-channel scalp electroencephalogram (EEG) from 25 successfully operated DRE patients were included. Secondly, time–frequency features by short-time Fourier transform (STFT), continuous wavelet transform (CWT), and superlets algorithm were extracted. Finally, the VGG-16 network was applied to automatically locate the epileptogenic zone. All three feature extraction methods achieved significant accuracy on the dataset. Using STFT for processing and combining it with VGG-16 for image classification achieved an average classification accuracy of 80.2% and a channel identification rate of 80.7% for epileptogenic zones. After processing with CWT, the accuracy increased to 81.7% and the epileptogenic zone channel recognition rate increased to 81.4%. After processing with the superlets method, the classification accuracy was further improved to 83.1%, and the epileptogenic zone channel recognition rate was increased to 83.3%. This marks the pioneering proposal of a systematic framework for non-invasive localization to the epileptogenic zone.

## 1. Introduction

Epilepsy is a chronic disease caused by abnormal discharge of brain neurons, resulting in impaired brain function and characterized by recurrent attacks and unpredictability [1]. Epilepsy often manifests as loss of consciousness and intense muscle spasms throughout the body, which can easily lead to disability or even death in patients [2]. At present, the number of epilepsy patients worldwide has exceeded 50 million, and the vast majority of patients live in underdeveloped areas [3]. At the same time, approximately one-third of epilepsy patients suffer from drug-resistant epilepsy (DRE), which makes it difficult to effectively control seizures through drug treatment and requires surgical removal of the epileptogenic zone for control [4]. Studies have shown that surgery is an important and safe and effective treatment for patients with DRE. Surgical treatment is superior to drug therapy in terms of long-term seizure control, likelihood of withdrawal, and improvement in quality of life [5].

Epilepsy surgery often requires the localization of the epileptogenic zone first, followed by treatment such as excision or radiofrequency fusion of the epileptogenic zone [6]. Therefore, effective localization of the epileptogenic zone is a prerequisite to ensure the success of the surgery. Currently, localization of epileptogenic regions in clinical practice still relies on the visual judgment of the physician through medical imaging methods such as electroencephalography, which is inefficient and highly dependent on the individual experience of the physician. There are numerous examples of surgical failure due to the incorrect localization of the epileptogenic zone. Therefore, if an automated epileptic zone localization algorithm can be developed, it will considerably improve processing efficiency and free physicians from the heavy burden of bioelectric signals.

High-frequency oscillation (HFO) has attracted attention due to its extremely high specificity, and studies have shown a strong correlation between HFO frequencies exceeding 80 Hz and epileptogenic zone [7]. It is mainly divided into 80–250 Hz Ripples rhythm, 250–500 Hz Fast Ripples rhythm, and very-high-frequency oscillations (VHFOs) above 600 Hz. HFO has extremely high specificity and mainly exists in the area of the epileptogenic zone, with little diffusion [8]. It has 100% specificity and is considered an effective biomarker for locating the epileptogenic zone [9]. It has important implications for the localization of epileptogenic regions and the prediction of surgical outcomes. Traditional HFO studies have focused on intracranial electroencephalography, but due to the limitations of invasive data collection, attention has been turned to non-invasive scalp electroencephalography. Matilde et al. used high-density EEG and magnetoencephalography (MEG) to locate the source of HFO, and achieved classification accuracies of 92% and 96%, respectively, based on the results of intracranial electroencephalography [10], which is expected to promote the development of non-invasive localization. However, although HFO is widely used in clinical practice and faces high expectations from researchers, there is actually no clear and convincing evidence to suggest that removing the area where HFO occurs can ensure that patients’ epilepsy will not recur [11]. A series of studies by Gliske et al. have shown that identifying HFO to locate and remove the epileptogenic zone has high limitations and further research is needed [12,13,14,15]. In addition to the high-frequency part, the sharp and spike waves during low-frequency EEG attacks are also considered important biomarkers [16], and the information contained in low-frequency EEG is also of great concern to people. In 2017, Adam Li et al. constructed a linear time invariant system for electrocorticogram (ECoG) data [17], which used mathematical methods to calculate vulnerability nodes to characterize the epileptogenic zone. The system results were highly similar to the actual results and did not require the observation of any seizure data. In addition to dynamic network analysis methods, more researchers choose to achieve automatic localization of the epileptogenic zone through preprocessing, feature extraction, and feature classification of EEG.

Due to the complexity of EEG signals, using some methods for feature extraction can uncover useful information from EEG data. Common feature extraction methods include entropy measurement [18], power spectral density [19], wavelet transform [20], deep convolutional autoencoder [21], etc. The extracted features are classified using machine learning algorithms such as k-nearest neighbor (KNN) [22], multilayer perceptron (MLP) [21], support vector machine (SVM) [20], and convolutional neural network (CNN) [23] to locate the epileptogenic zone. Although such epilepsy localization methods are widely used and have achieved excellent results, there are some limits. Firstly, the feature extraction or model structure design in many studies are selected and designed by researchers based on the datasets in their papers. However, due to the small number of patients or single case types in the datasets themselves, the good results obtained from the paper datasets may not be repeatable in real clinical situations. The second reason is that many current studies use invasive intracranial electroencephalography, which not only poses a risk of complications due to its invasiveness, but also ignores other areas due to its limited spatial resolution, resulting in missing the epileptogenic zone. Therefore, it is important to develop non-invasive methods for the localization of the epileptogenic zone. At present, there is relatively little work on non-invasive epileptogenic zone prediction, and poor prediction results make it difficult to guide clinical diagnosis.

Previous non-invasive epilepsy localization often used EEG or MEG from the scalp to infer the source imaging of the electrical activity of epileptic seizures in the brain. This study also used scalp EEG as experimental data. Traditional methods often analyze EEG in the frequency domain [24,25], time domain [26,27,28,29], or time–frequency domain [30,31] to capture more information, and these methods are widely used and effective. This article uses time–frequency analysis tools such as short-time Fourier transform (STFT), continuous wavelet transform (CWT), and superlets to perform time–frequency domain analysis on EEG data. In order to automatically and better capture time–frequency domain information, we plotted the time–frequency domain analysis results in the form of a heatmap and trained and classified these maps using Visual Geometry Group (VGG-16) convolutional neural network to distinguish between EEG information from the epileptogenic zone and non-epileptogenic zone. This approach can effectively utilize the ability of deep neural networks to mine information and achieve automatic classification of epileptogenic zone. By placing electrodes on the scalp to collect and analyze multiple channels of EEG signals, the epileptogenic zone can be predicted. This can reduce the damage of invasive collection to the patient’s brain, reduce the difficulty of collection, and simplify the diagnosis and treatment process of epilepsy, while ensuring the cure rate and improving efficiency [32]. Another highlight of this article is that the dataset used in this study has a large amount of data and a wide coverage area. At the same time, the network model can automatically extract features for classification, without the need to design complex network structures for the dataset. This allows us to maintain better performance and excellent recognition accuracy with more accurate time–frequency features. This study is aimed at clinical practice and constructs a unified standardized framework using the most widely used scalp EEG data to easily locate potential epileptogenic zones.

## 2. Materials and Methods

### 2.1. Dataset Description

In this paper, we collected scalp EEG data collected during 60 seizures in 25 patients at the Second Hospital of Lanzhou University. All the 25 patients were examined and diagnosed by two neurologists. The number of electrodes placed was 16, the sampling time was 10 s, and the sampling frequency was 500 Hz. Please see Table 1 for specific demographic information. Then, we processed the collected EEG data using the standardized low-resolution brain electromagnetic tomography (sLORETA) source imaging method to obtain the brain electrical activity with 148 channels. The epileptogenic zone channels of these 25 patients were determined by clinical doctors, and there was no recurrence after surgery, indicating that the determination of epileptogenic zone channel location in our data is correct or has practical guidance significance for clinical surgery. When using these data, the model decomposes the newly obtained EEG data into 148 single-channel EEG data, which are divided into epileptogenic zone and non-epileptogenic zone categories according to clinical annotations. In this way, we obtained a total of 8880 EEG data, including 717 EEG data from the focal channels and the remaining 8163 EEG data from the non-focal channels. The amount of data we used far exceeds that of some studies using publicly available datasets. This study was approved by the Ethics Committee of the Second Hospital of Lanzhou University (approval No. 2023A-765).

### 2.2. EEG Source Imaging

**Figure 1:**
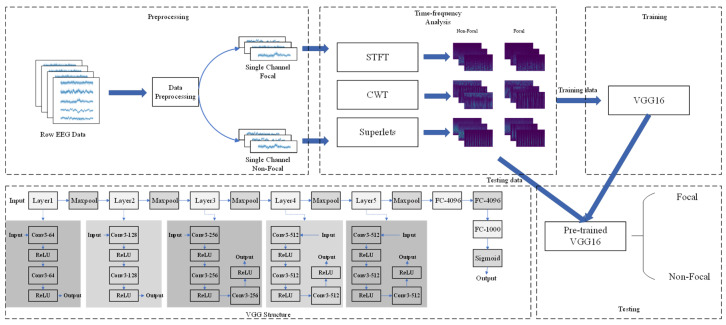
Model processing and VGG-16 structure diagram. Conv: convolutional layers; ReLU: activation function. FC: full connectivity layer.

In this study, a non-invasive epilepsy localization method was constructed by integrating sLORETA source imaging, time–frequency analysis, and VGG-16 deep learning. Firstly, 16-channel scalp EEG from 25 successfully operated DRE patients were included. Secondly, STFT time–frequency features, CWT, and superlets algorithm were extracted. Finally, the VGG-16 network was applied to automatically locate the epileptogenic zone. The structure diagram of this study is shown in Figure 1.

EEG source imaging technology is a commonly used non-invasive method for EEG analysis. Using source imaging, we can use very few non-invasive electrodes, combine the collected brain electrical activity (EEG or MEG) with brain anatomical information, and use some mathematical and computational models to infer the source location of epileptic seizures in the brain [33]. The national standard 10–20 system is currently the standard detection configuration used in clinical practice. To adapt to the actual clinical situation, this article uses 16-channel scalp EEG data. Source imaging technology essentially infers internal electric field distribution based on known external electric fields. Due to volume conduction effects [34], multi-channel scalp electroencephalography is required to reconstruct intracranial neural activity and achieve precise localization. We use the sLORETA source imaging method. Compared with other methods, it is simpler and has acceptable accuracy, higher AUC, and lower positioning error rate [35,36,37]. The sLORETA defines the scalp electrical potentials as follows:

(1) Φ=KJ+c1

where $\Phi \in R^{N_{E}\times 1}$ is a vector containing scalp electric potentials measured at $N_{E}$ cephalic electrodes, with respect to a common, arbitrary reference electrode located anywhere on the body. The primary current density $J\in R^{{(3N}_{V})\times 1}$ is defined as follows:

(2) $$J={\left(J_{1}^{T},J_{2}^{T},J_{3}^{T},\ldots ,J_{N_{v}}^{T}\right)}^{T}$$

where component $J_{l}^{T}$ is a vector containing three unknown dipole moments. T represents transposition. $K\in R^{N_{E}\times (3N_{E})}$ refers to the matrix of lead filed, where $k_{ij}=(k_{i,l}^{x},k_{i,l}^{y},k_{i,l}^{z})$ is the scalp electric potential at the $i^{th}$ electrode due to a unit strength x−,y−,z− oriented dipole at the $l^{th}$ voxel. c is an arbitrary constant which embodies the fact that the electric potential is determined up to an arbitrary constant; $1\in R^{N_{E}\times 1}$ is a vector of ones. The parameter c allows the use of any reference for the lead field and the measurements. The function of interest is

(3) $$F={\left\|\Phi -KJ-c\left.1\right\|\right.}^{2}+{\alpha \left\|\left.J\right\|\right.}^{2}$$

where α≥0 is a regularization parameter. This function is to be minimized with respect to J and c, for given K, Φ, and α.

(4) $$\hat{J}=\underset{J,c}{\min}F=\underset{J,c}{\min}\left({\left\|\Phi -KJ-c\left.1\right\|\right.}^{2}+{\alpha \left\|\left.J\right\|\right.}^{2}\right)$$

Finally, $\hat{J}$ should be further scaled to become the source estimation of sLORETA. For further details, please refer to the study [37]. During the sLORETA analysis, we used the boundary element methods (BEMs) to build a realistic head model, which was processed using MATLAB (MATLAB 2023b)’s brainstorming toolbox. Based on the brain regions defined by the destrieux atlas, 74 × 2 (left and right hemispheres) = 148 brain channels were analyzed for brain power. Then, we labeled the surgical area as the epileptogenic zone, and the rest as the non-epileptogenic zone. Since the data used are from patients with successful surgery, these annotations can be considered true and effective.

### 2.3. Short-Time Fourier Transform

STFT is developed on the basis of fast Fourier transform (FFT). Fourier transform can synthesize or decompose stationary signals, but it cannot perform time–frequency analysis on non-stationary signals because it ignores the time information of the signal. STFT involves windowing the signal, decomposing it into several equally long small windows, approximating the signal within the window as a stationary signal, and then using Fourier transform to analyze the signal characteristics and obtain its spectral information [38]. EEG is a typical non-stationary signal [39], so STFT is very suitable for processing EEG data.

The STFT is defined as follows [40]:

(5) $$STFT\left\{x\left(t\right)\right\}\left(\tau ,\omega \right)=\int_{-\infty }^{\infty }x\left(t\right)\omega \left(t-\tau \right)e^{-i\omega t}dt$$

where $x\left(t\right)$ is the signal to be analyzed, and ω(t) is a short-term window function. This study uses the STFT tool in the Scipy package to perform STFT with a window length of 500 samples.

### 2.4. Continuous Wavelet Transform

Wavelet transform is a classic time–frequency analysis method that can decompose signals into the superposition of different wavelets [41], effectively extract local time–frequency features of signals, and is well suited for processing non-stationary signals [42]. It can also accurately capture changes and sudden event information in signals, making it suitable for processing EEG and other data. Discrete wavelet transform has high computational efficiency and is suitable for signal compression, denoising, and feature extraction. The CWT has high time–frequency resolution and can provide detailed instantaneous frequency and phase transformations of signals. It can better capture local characteristics of signals and is suitable for non-stationary signal analysis.

The CWT is defined as follows [43]:

(6) $$W\left(a,b\right)=\frac{1}{\sqrt{a}}\int_{-\infty }^{+\infty }x\left(t\right)\cdot \psi \left(\frac{t-b}{a}\right)dt$$

where W(a,b) is the wavelet coefficient, x(t) is the input signal to be transformed, t is time, ψ is the wavelet basis function, a is the scale parameter used to determine the center frequency of the basis function, and b is the translation parameter that controls the position of the wavelet on the time axis.

Wavelet transform uses a series of wavelet basis functions to match and decompose the original signal and generates corresponding wavelet functions using different wavelet basis functions to be applicable to different signal analysis scenarios. Morlet wavelets are a family of wavelets in CWT, defined as follows [44]:

(7) $$\psi \left(t\right)=e^{j2\pi \omega_{c}t}e^{{-t}^{2}/2\sigma^{2}}$$

where t is time, $\omega_{c}$ is the average of the lower cut-off frequency and upper cut-off frequency of Morlet, σ is a parameter that controls the scale of the Gaussian kernel $e^{{-t}^{2}/2\sigma^{2}}$, and the Morlet morther wavelet is the Gaussian kernel weighted sine wave.

Morlet wavelets have many advantages, such as high concentration in both time and frequency domains, which can accurately capture the transformation information of signals. On the other hand, the wavelet also has high frequency resolution and can handle high-frequency signals well, making Morlet very suitable for processing EEG data. This article will use the open-source Python 3.12 library PyWavelets to perform continuous wavelet transform on the original signal using Morlet wavelets, and plot the returned time–frequency analysis results into a time–frequency graph. The time–frequency diagram intuitively displays the frequency variation of a signal over time, with the horizontal axis representing time and the vertical axis representing frequency. The color depth of the image represents the intensity of the amplitude.

### 2.5. Superlets Transform

STFT or CWT can only optimize time resolution or frequency resolution or find suboptimal equilibrium, and it is difficult to localize in both time and frequency. Vasile et al. proposed a time–frequency analysis method called superlets, which uses wavelet sets with increasingly limited bandwidth to achieve frequency resolution in higher frequency bands while maintaining good temporal resolution of individual wavelets. Experiments have shown that superlets perform well in synthesizing data and brain signals recorded by humans and rodents, and can effectively resolve high-frequency components.

The author defined a superlet (SL):

(8) $${SL}_{f,o}=\left\{\psi_{f,c}|c=c_{1},c_{2},\ldots ,c_{o}\right\}$$

It consists of a set of wavelets with a fixed center frequency f, where o is the order of the superwavelet, and $c_{1},c_{2},...,c_{o}$ is the period of each wavelet in the set. From this, it can be seen that an SL is a finite set of o wavelets with different bandwidths and the same center frequency f. The period of the wavelets in this set can be determined by multiplication or addition. When using multiplication,

(9) $$c_{i}=i\;*\;c_{1}\;$$

When using addition,

(10) $$c_{i}=c_{1}+i-1$$

**Figure 2:**
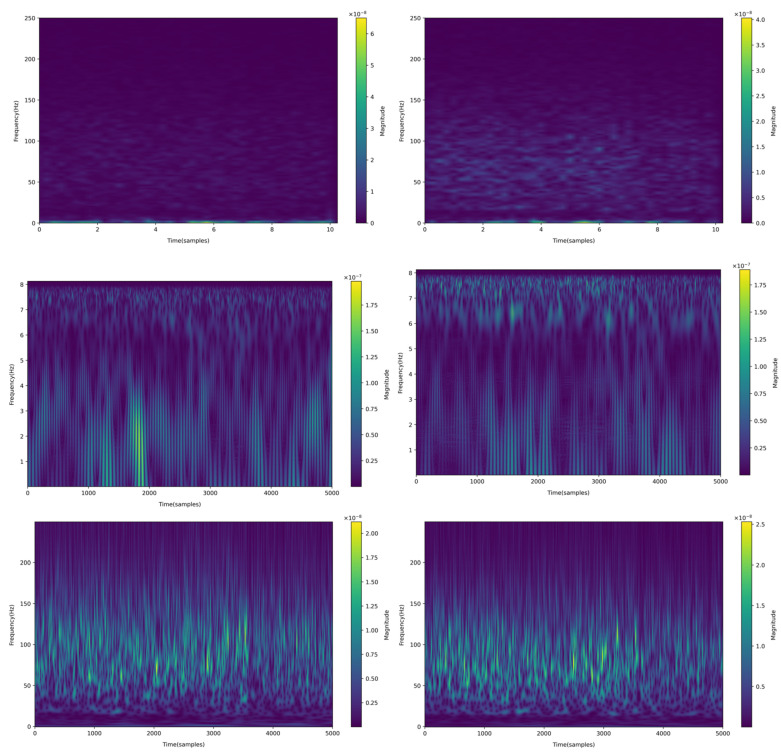
From top to bottom are the processing results of STFT, CWT, and superlets, and from left to right are the processing results of epileptogenic zone and non-epileptogenic zone.

The response of SL to signal x is defined as the GM of a single wavelet response in the set:

(11) $$R\left[SL_{f,o}\right]=\sqrt[{o}]{\prod_{i=1}^{o}R\left[\psi_{f,c_{i}}\right]}$$

where $R\left[SL_{f,o}\right]$ is the response of wavelet i to the signal. Taking Morlet wavelet as an example,

(12) $$R\left[\psi_{f,c_{i}}\right]=\sqrt{2}\;\cdot \;x\;*\;\psi_{f,\;c_{i}}$$

where x is the signal, and ∗ is the convolution calculation. An ultra wavelet is essentially an estimation of the oscillation packet of a signal at the center frequency f, and the signal amplitude is estimated by calculating the corresponding amplitudes of each wavelet; When calculating the scale of the signal, only the size of SL needs to be simply squared. The calculation of signal SL transform is similar to CWT, except that SL uses multiple wavelets. When the SL is of order 1, it is actually a continuous wavelet transform. When the order of SLT is greater than one, it has less redundancy compared to the corresponding CWT and can represent the signal more clearly. The example time–frequency spectra of source EEG estimated by STFT, CWT, and superlets methods are shown in Figure 2.

### 2.6. VGG Convolutional Neural Network

The VGG convolutional neural network was proposed by the University of Oxford in 2014 and developed from the LeNet and AlexNet networks. Compared to AlexNet, it has smaller convolution kernels (3 × 3 convolution kernels and 2 × 2 pooling kernels) and larger network depths (including 16 convolutional and fully connected layers VGG-16 and 19 convolutional and fully connected layers VGG-19), successfully proving that larger network depths are beneficial for improving network performance. The input image size of VGG is a constant 224 × 224. Through several convolutional pooling layers and three fully connected layers, various results are mapped to [0,1] in the form of probability distributions using a soft max function. Compared with previous networks, smaller convolutional kernels require less parameters for the model, while larger network depths have stronger feature extraction capabilities than shallow networks, which is a major advancement for VGG compared to other graph classification networks. Experiments have shown that VGG-16 and VGG-19 achieved the best top-1 error rates of 25.6% and 25.5%, as well as top-5 error rates of 8.1% and 8.0%, respectively, in single scale evaluation. In the multi-size evaluation, both VGG-16 and VGG-19 achieved a top-1 error rate of 24.8% and a top-5 error rate of 7.5%.

In this study, VGG-16 was used for the classification task. The model consists of 6 block structures with a total of 13 convolutional layers, 5 pooling layers, and 3 fully connected layers, with all activation units being ReLU activation functions. The cross-entropy loss function and Adam optimizer were used for feedback during design, and dropout layer was added in the network to avoid overfitting. In the data preprocessing stage, each image needed to be processed to a size of 224 × 224, and all epileptogenic and non-epileptogenic regions’ data needed to be placed in two separate folders for the algorithm to label the images at readout. The images and their labels were fed into the network for training. The size ratio of the training and test sets was 7:1, uniformly set to 80 epochs, and each batch size was 16.

## 3. Results

The proposed method was implemented on a workstation with an RTX 3090 (24 GB) GPU, 14 vCPU Intel (R) Xeon (R) Platinum 8362 CPU @ 2.80 GHz, and 45 GB of RAM using the Ubuntu 18.04, Python 3.8, and CUDA11.04 environment on the PaddlePaddle framework on a cloud server. The STFT was implemented using the Scipy package, the CWT was implemented using the PyWavelets package, and the superlets was modified using the source code provided by the original paper author. The VGG image classification section is written using the paddle framework. After time–frequency domain analysis of the raw data, there were a total of 8163 non-epileptogenic zone channel images and 717 epileptogenic zone channel images in the dataset. In this paper, we performed down sampling on the non-epileptic channel images during training, and randomly selected 717 non-epileptic channel images and all epileptogenic channel images to form a new dataset during each training epoch. The ratio of the training set to the test set was 7:1. Considering the data partitioning method and data distribution, the validation set was not partitioned.

**Figure 3:**
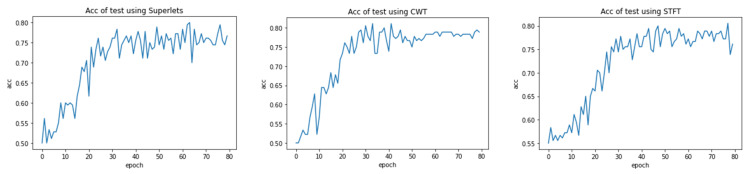
The accuracy variation of three time–frequency analysis methods on the test set. As the epoch increases, the average accuracy tends to stabilize.

Due to the long time required for a single training session, to improve efficiency, this paper did not adopt the traditional cross-validation method. Instead, before each training session, images were randomly sampled from non-epileptogenic zone channels and the dataset was divided. The result with the highest classification accuracy on the test set during this training process was used as the training result. The accuracy variation of three time–frequency features on the test set is shown in Figure 3. The preliminary experiment in Figure 3 found that the accuracy tended to stabilize after 80 epochs, so the maximum epoch of the neural network was set to 80 and the batch size was 16.

Each epoch of training evaluates the performance of the model on the test set, and the best result is taken as the performance of the model. The average over multiple training sessions is used as the final performance of the model. The model uses accuracy, precision, recall, negative class sample precision, and negative class sample recall as evaluation criteria. In Equation (13), TP represents true positive, FP is false positive, TN is true negative, and FN is false negative. The positive and negative class accuracy and recall reflect the model’s ability to recognize two types of samples, avoiding the “false impression” of high accuracy caused by the model’s “bias” toward a certain type of sample.

(13) $$\begin{array}{c} Accuracy=\frac{TP+TN}{TP+FP+FN+TN}\\ Precision=\frac{TP}{TP+FP}\\ Recall=\frac{TP}{TP+FN}\\ N-recall=\frac{TN}{FP+TN}\\ N-preciosin=\frac{TN}{FN+TN} \end{array}$$

The performance indicators obtained through STFT, CWT, and superlets are shown in Table 2. It can be seen that all three time–frequency analysis methods achieved a classification accuracy of over 80%, with the superlets method achieving an average classification accuracy of 83.1% and an epileptogenic zone sample recognition rate of 83.3% when processing EEG signals. From the comparison of the results of the three methods, it can be seen that the higher the accuracy of the time–frequency analysis method used, the higher the classification accuracy of the model. Especially after using superlets, an accuracy of 83.1% was achieved, which is superior to some previous research results (as shown in Table 3 [45,46,47,48,49]. In contrast, the proposed method uses time–frequency analysis tools to extract time–frequency information and then uses deep neural networks to further mine and classify the information, which consumes less manpower and computational resources and has excellent performance.

## 4. Discussion

Due to the heavy workload, low efficiency, and relatively low classification accuracy of current methods for locating epileptogenic zones in clinical practice, automatic and accurate classification of epileptogenic zones based on EEG signals will greatly facilitate the treatment of epilepsy. The main advantage of this paper over previous results is as follows:

(1) Excellent model performance and relatively high classification accuracy.

When extracting signal features, the time–frequency map of STFT shows that more energy is concentrated in the low-frequency part, and excessive attention to the high-frequency part may lead to a decrease in accuracy. When performing continuous wavelet transform on EEG signals, we use Morlet wavelets for wavelet transform. The time–frequency analysis section is concentrated in the low-frequency range of 0.81–8.1 Hz, resulting in a slight improvement in accuracy compared to the STFT section. Superlets is a novel time–frequency analysis method that is particularly suitable for time–frequency analysis related to EEG. It has high accuracy and the highest precision in time–frequency analysis.

Three time–frequency analysis tools combined with VGG-16 were used for automatic classification of the epileptogenic zone, and all three methods achieved a classification accuracy of over 80%. Especially after using superlets, an accuracy of 83.1% was achieved, which is superior to some previous research results (as shown in Table 2). Additionally, the method proposed in this article uses time–frequency analysis tools to extract time–frequency information, and then uses deep neural networks for further mining and classification of information, which consumes less manpower and computing resources and has excellent performance.

(2) Using non-invasive methods to locate the epileptogenic zone.

In terms of locating the epileptogenic zone, previous researchers mostly used iEEG as analysis data. The main reason is that iEEG has higher temporal and spatial resolution, high signal-to-noise ratio, and better locality, which can more accurately locate the epileptogenic zone and is the gold standard for clinical epilepsy diagnosis. It can also record the continuous EEG data of patients for hours or even days, which can provide a lot of valuable information for dissecting the epilepsy onset area [50]. But at the same time, iEEG has many limitations. For example, as an invasive method of EEG acquisition, iEEG may not only cause complications due to its invasiveness, but also overlook other areas and miss the epileptogenic zone area due to its limited resolution [51,52].

This article uses the patient’s scalp EEG, which is a non-invasive EEG acquisition method that can effectively avoid the shortcomings of iEEG. Using scalp EEG can help us locate the potential epileptogenic zone in advance, guide the accurate implantation position of intracranial electrodes, and minimize the risks associated with invasive surgery as much as possible. Therefore, the development of non-invasive epileptogenic zone localization is of great significance. This article constructs a model for non-invasive epileptogenic zone localization and achieves good results, which, to some extent, promotes the development of non-invasive epileptogenic zone localization.

(3) There are a large number of datasets with diverse categories.

The article used real EEG data from clinical patients and collected 60 episodes of scalp EEG during the attack period from 25 patients. Compared with some results using publicly available datasets [21], this study has a larger number of patients, a larger amount of data, and a more diverse range of patient categories, which can better adapt to real clinical environments and achieve better results in clinical practice.

The epileptogenic zone labeling data used in this article are all provided by professional clinical doctors and validated through surgical results, ensuring that the labeled epileptogenic zone channels are true and accurate. Due to factors such as data volume, some previous studies also used patient data from surgical failures [53], which means that epileptogenic zone labeling is likely to be incorrect. Using incorrect labels to evaluate predictive performance is highly inaccurate. The data used in this article are all from successful surgical patients, which can effectively remove the influence of incorrect labels on the model and is more suitable for guiding the actual surgical area in clinical practice.

There are also some limits in this article, such as the gap between the classification accuracy obtained in this article and the current optimal results, and due to time constraints, this article did not consider the details of the model extensively, such as the time–frequency analysis parameters such as the STFT window scale, as well as the classification model parameters such as batch size and learning rate for VGG-16 processing data, which were not further modified and explored. Herein, some other light weight classifiers like SqueezeNet and ResNet are also discussed, and the results are shown in Table 4.

In fact, our original result in Table 2 is the best result after comparative study; however, some of the more complex and updated neural networks have not been fully included. The core innovation of this study is to propose a novel non-invasive localization framework, namely, time–frequency spectrograms and deep learning. As a result, some of the more complex and updated neural networks have not been fully included, and we will introduce more networks and more data, including multi-center data, in a later work to further advance the accurate diagnosis and treatment of refractory epilepsy. It can be concluded that the proposed method is worthy of further research and promotion. It is expected that, in future work, the current problems can be further corrected and optimized to further improve the performance of the model.

## Figures and Tables

**Table 1:** Demographic and clinical information of the patient cohort.

#	Sex	Age at Surgery	Age Epilepsy Onset	Etiology	Engel Grading	Num. of EZ	Num. of Seizures
1	M	31	27	Unknow	Engel I	5	1
2	M	23	3	FCD	Engel I	14	2
3	F	12	11	Unknow	Engel I	11	4
4	M	28	20	Unknow	Engel I	13	4
5	F	8	7	Unknow	Engel I	10	2
6	F	28	12	Unknow	Engel I	12	3
7	M	36	18	Unknow	Engel I	12	2
8	M	23	16	Unknow	Engel I	11	2
9	M	15	3	FCD	Engel I	18	4
10	M	30	25	Unknow	Engel I	10	2
11	F	26	6	Unknow	Engel I	11	3
12	M	22	7	Unknow	Engel I	9	3
13	F	16	13	Unknow	Engel I	7	2
14	M	12	7	Unknow	Engel I	10	1
15	M	22	20	FCD	Engel I	5	2
16	F	35	16	Unknow	Engel I	15	4
17	M	28	4	Unknow	Engel I	10	1
18	F	31	5	FCD	Engel I	6	3
19	F	26	7	Unknow	Engel I	6	3
20	M	37	35	FCD	Engel I	15	2
21	F	20	18	Unknow	Engel I	25	2
22	M	18	10	FCD	Engel I	10	2
23	F	31	21	Tuberous sclerosis	Engel I	11	3
24	M	26	1	Unknow	Engel I	28	2
25	M	22	9	Unknow	Engel I	5	1

FCD: focal cortical dysplasia. EZ: epileptogenic zone.

**Table 2:** Model performance indicators.

Time–Frequency Spectrum	Accuracy	Precision	Recall	N-Precision	N-Recall
STFT	80.2%	80.1%	80.7%	80.7%	79.6%
CWT	81.7%	82.1%	81.4%	81.8%	81.7%
superlets	83.1%	83.0%	83.3%	83.4%	82.8%

**Table 3:** Comparison results in locating epileptogenic zone.

Author (Year)	Data	Extraction Methods	Classifier	Accuracy
Adam Li et al. (2022) [45]	iEEG	neural fragility of the iEEG network	/	76%
Jeong-Won et al. (2022) [46]	iEEG	multi-model MRI features	msResNet	75%
Paige M.Murphy et al. (2017) [47]	iEEG	HFO	/	70%
Bernd et al. (2021) [48]	HD-EEG	spike detection and clustering	/	55–71%
Davide et al. (2020) [49]	PET, MEG, EEG-fMRI, HR-EEG	/	/	80%
**Ours**	**Scalp EEG**	**STFT**	**VGG-16**	**80.20%**
		**CWT**		**81.70%**
		**superlets**		**83.10%**

**Table 4:** The classification results of SqueezeNet and ResNet.

Deep Learning	Time–Frequency Feature	Accuracy	Precision	Recall	N-Precision	N-Recall
SqueezeNet	STFT	79.1%	78.1%	79.7%	80.2%	78.4%
	CWT	79.8%	81.1%	79.2%	80.2%	80.3%
	Superlets	80.3%	81.4%	82.2%	81.8%	81.1%
ResNet	STFT	79.3%	78.2%	79.5%	79.8%	78.8%
	CWT	79.9%	80.8%	80.0%	80.4%	80.1%
	Superlets	80.6%	81.1%	81.8%	81.6%	80.4%

## Data Availability

The code and some example image data supporting the findings of this study have been uploaded to Github: https://github.com/wyl1994/data-and-code-for-our-paper-Noninvasive-localization-of-epileptogenic-zone...- (accessed on 5 October 2024). For more data, please contact the corresponding author of this article.
